# Childhood trauma in bipolar disorder

**DOI:** 10.1177/0004867413516681

**Published:** 2014-06

**Authors:** Stuart Watson, Peter Gallagher, Dominic Dougall, Richard Porter, Joanna Moncrieff, I Nicol Ferrier, Allan H Young

**Affiliations:** 1 The Institute for Neuroscience, Newcastle University, Newcastle, UK; 2 Faculty of Brain Sciences, University College London, London, UK; 3 Department of Psychological Medicine, University of Otago, Christchurch, New Zealand; 4 Centre for Affective Disorders, Institute of Psychiatry, Kings College London, London, UK

**Keywords:** Bipolar disorder, childhood trauma, depression, emotional neglect

## Abstract

**Objective:**

There has been little investigation of early trauma in bipolar disorder despite evidence that stress impacts on the course of this illness. We aimed to compare the rates of childhood trauma in adults with bipolar disorder to a healthy control group, and to investigate the impact of childhood trauma on the clinical course of bipolar disorder.

**Methods:**

Retrospective assessment of childhood trauma was conducted using the Childhood Trauma Questionnaire (CTQ) in 60 outpatients with bipolar disorder being treated for a depressive episode and 55 control participants across two centres in north-east England and New Zealand.

**Results:**

Significantly higher rates of childhood trauma were observed in patients with bipolar I and bipolar II disorder compared to controls. Logistic regression, controlling for age and sex, identified emotional neglect to be the only significant CTQ subscale associated with a diagnosis of bipolar disorder. Childhood history of sexual abuse was not a significant predictor. Associations with clinical severity or course were less clear.

**Conclusions:**

Childhood emotional neglect appears to be significantly associated with bipolar disorder. Limitations include the relatively small sample size, which potentially increases the risk of type II errors. Replication of this study is required, with further investigation into the neurobiological consequences of childhood trauma, particularly emotional neglect.

## Introduction

The high prevalence and incidence (Merikangas et al., 2011), chronicity of symptoms (Judd et al., 2002, 2003), and psychosocial impairment (Judd et al., 2005) of bipolar disorder underlines the need to establish its aetiological and risk factors. Bipolar disorder is highly heritable (McGuffin et al., 2003); psychosocial stress also appears to increase the likelihood of first and possibly subsequent episodes (Etain et al., 2008; Post, 1992). Childhood trauma is a recognised indicator of poor prognosis in major depressive disorder (Douglas and Porter, 2012; Nanni et al., 2012) but, in bipolar disorder, whilst the impact of stressors in adulthood on the course of illness has been investigated (Cohen et al., 2004; Paykel, 2003), the impact of early trauma has been relatively neglected. One study has shown that early parental loss is more common (Agid et al., 1999), whilst others have shown that childhood stressful life events are less common (Horesh et al., 2011) or as common (Horesh and Iancu, 2010) in bipolar disorder compared with healthy controls. Children and adolescents with bipolar disorder have been shown to be exposed to more negative life events and less positive events compared to controls (Romero et al., 2009), although interestingly, a recent paper suggested that the link between stressful events and bipolar disorder may be a consequence of the illness (Hosang et al., 2012). Studies using the Childhood Trauma Questionnaire (CTQ) (Bernstein et al., 2003) have reported a higher rate of childhood trauma (Fowke et al., 2012), particularly emotional abuse (Etain et al., 2010), in bipolar disorder. Retrospectively reported childhood abuse has been associated with an adverse illness course (Garno et al., 2005; Leverich et al., 2002), more depressive episodes (Garno et al., 2005), greater severity of mania (Garno et al., 2005; Leverich et al., 2002), with earlier onset (Carballo et al., 2008; Garno et al., 2005; Leverich et al., 2002), suicidal ideation (Carballo et al., 2008; Leverich et al., 2002), substance abuse (Brown et al., 2005; Carballo et al., 2008), and with impaired performance on tests of neuropsychological function (Savitz et al., 2008). However, interpretation of these findings is limited by the clinical and methodological heterogeneity of these studies (Daruy-Filho et al., 2011).

In this study, childhood trauma, as measured by the CTQ, was compared in a sample of people with bipolar disorder recruited for a randomised trial (Watson et al., 2012) and in a healthy control group. It was predicted that higher CTQ scores would be associated with a diagnosis of bipolar disorder and secondly, that childhood trauma would be associated with measures of clinical severity.

## Methods

### Sample

This analysis uses baseline assessment data from a randomised placebo-controlled trial of mifepristone treatment in bipolar depression (Watson et al., 2012). The study was carried out in two centres, Newcastle University in the north-east of England and Otago University in Christchurch, New Zealand.

The main inclusion criterion was a diagnosis of bipolar disorder current episode depressed, confirmed with the Structured Clinical Interview for DSM-IV (SCID) (First et al., 1997). Additional inclusion criteria were: age between 18 and 65 years, stable medication for a minimum of 4 weeks, the ability to provide informed consent and the ability to adequately understand both written and verbal English. Both men and women were eligible.

Potential participants were excluded if they fulfilled criteria for substance abuse or dependence (First et al., 1997), were pregnant, suffered significant medical illness which would render recruitment into the clinical trial unsafe (such as: suffered head trauma with persistent loss of consciousness, a neurological disorder or uncompensated endocrine disorder). A co-morbid axis II diagnosis was not an exclusion criterion. After a complete description of the study, written informed consent was obtained from all participants. The study received full approval from the local ethics committee.

Participants were recruited from outpatient clinics allied to the respective centres. Sixty patients were randomized over a 5-year period from October 2004, of which 31 patients met SCID criteria for bipolar I and 25 the criteria for bipolar II. A cohort of 55 age- and sex-matched comparators, who were SCID confirmed as having no current or past history of an axis I disorder, was concurrently locally recruited.

### Assessment

After an initial screening visit, baseline data was collected by trained psychiatrists with full history, case note and medication review. The data included demographic and clinical characteristics of sex, age, body mass index (BMI), pre-morbid IQ measured by the National Adult Reading Test (NART) (Nelson and Willison, 1991) and number of years of education. Measures which may indicate clinical severity included: the 17-item version of the Hamilton Depression Rating Scale (HDRS-17) (Hamilton, 1960); diagnosis of DSM-IV melancholia; length of the current depressive episode (weeks); number of previous hospitalisations; current alcohol intake (standard UK alcohol units per week); diagnosis of rapid cycling bipolar disorder; history of attempted suicide; any form of current suicidal ideation reported to the assessor.

The childhood trauma questionnaire (CTQ) was also completed. The CTQ is a validated 28-item self-report questionnaire used to provide a retrospective measure of childhood trauma (Bernstein et al., 2003). It uses a five-point Likert-type scale. Twenty-five of the CTQ questions are split into five subscales of maltreatment: emotional abuse, physical abuse, sexual abuse, emotional neglect and physical neglect. The other three questions are used for detecting ‘false-negative’ answers involving a minimisation/denial scale.

### Statistical analyses

Distributions of CTQ scores did not meet the assumption required for parametric analysis. Where appropriate, the bipolar group was divided into SCID (First et al., 1997) determined bipolar I and bipolar II subgroups. Chi-squared (χ^2^) test was used to compare sex distribution across bipolar and control groups. Age and BMI were normally distributed and were compared across bipolar and control groups using the independent samples *t*-test. The Mann–Whitney *U*-test and Spearman’s rank order correlations were used for all other comparisons of continuous variables.

CTQ subscales for pooled bipolar and control data were examined using Spearman’s rho to identify significant correlations between subscales to determine suitability for inclusion in a regression analysis. Spearman’s rho was also used to examine relationships between CTQ subscale scores and the demographic variables, age, pre-morbid IQ (NART score) and years of education. CTQ subscale scores were compared between males and females. Step forward logistic regression of CTQ total scores and relevant demographic variables was performed to examine the overall relationship between trauma and bipolar disorder, with the binary outcome variable of bipolar or control group. A separate step forward logistic regression of the relevant CTQ subscales and demographic variables was performed to explore for relationships according to types of trauma.

## Results

Patients and controls were matched for age, sex, pre-morbid IQ (NART score) and years of education, as reported in Table 1.

**Table 1.** Demographic and clinical characteristics of the bipolar group and control group.

	Bipolar patients % or mean (SD)	Controls % or mean (SD)	Comparison (*p*)
Male (%)	53.3	54.5	χ^2^ = 0.02 (0.896)
Age (mean years)	47.9 (9.4)	45.1 (13.1)	*t* = 1.3 (0.193)
BMI	29.8 (6.2)	26.0 (3.7)	*t* = 3.0 (0.004)
NART IQ	110.6 (10.5)	113.3 (11.3)	*U* = 1152.5 (0.089)
Years of education	14.7 (3.3)	14.8 (4.3)	*U* = 917.0 (0.554)

BMI: body mass index; NART IQ: National Adult Reading Test IQ.

A diagnosis of bipolar disorder was found to be significantly associated with a greater total CTQ score (Table 2). All subscale scores were significantly higher in the bipolar group, apart from sexual abuse. Similar results were found when the analysis was restricted to those with a diagnosis of bipolar I. In participants diagnosed with bipolar II, CTQ total, emotional abuse, emotional neglect and physical neglect scores were significantly greater than controls.

**Table 2.** CTQ scores in bipolar groups compared to controls.^a^

	All bipolar *N* = 60^b^				Bipolar I *N* = 31^b,c^				Bipolar II *N* = 25^b,c^				Control *N* = 55^%^	
			Comparison				Comparison				Comparison			
	CTQ	(SD)	*U*	*p*	CTQ	(SD)	*U*	*p*	CTQ	(SD)	*U*	*p*	CTQ	(SD)
CTQ total	44.4	(19.1)	490.0	< 0.001	43.6	(20.6)	280.5	0.004	41.1	(13.1)	203.0	0.003	31.2	(8.0)
Emotional abuse	10.4	(5.4)	780.0	< 0.001	10.2	(5.8)	470.0	0.012	9.7	(4.3)	294.0	0.005	6.8	(2.8)
Physical abuse	7.5	(4.4)	903.0	0.005	7.9	(4.9)	461.0	0.005	6.0	(2.1)	439.5	0.387	5.4	(1.3)
Sexual abuse	7.7	(5.4)	1092.5	0.131	7.9	(4.9)	584.5	0.182	7.1	(4.1)	428.5	0.224	6.2	(3.1)
Emotional neglect	12.4	(6.0)	767.0	< 0.001	12.7	(7.0)	459.5	0.011	11.1	(4.2)	299.0	0.008	8.2	(3.5)
Physical neglect	7.9	(3.8)	787.5	< 0.001	8.1	(4.2)	446.0	0.002	7.2	(3.0)	306.5	0.003	5.7	(1.7)

a Table showing mean and SD of CTQ scores in different subject groups with a comparison using Mann–Whitney *U*-test of CTQ scores between the bipolar groups (all bipolar patients and those with a diagnosis of bipolar I or bipolar II) with controls.

b Numbers vary due to the incomplete return of CTQs: all bipolar, *N* = 49–57; bipolar I, *N* = 25–31; bipolar II, *N* = 20–22; control, *N* = 39–45.

c Four participants with bipolar disorder were not sub-classified as either bipolar I or II.

CTQ: Childhood Trauma Questionnaire.

Table 3 shows that in bipolar patients, CTQ scores did not differ between those with and those without suicidal ideation, although scores for the emotional neglect subscale showed a trend towards significance. Participants with a diagnosis of DSM-IV melancholia had significantly higher CTQ total scores, and significantly higher emotional neglect and emotional abuse scores than those without. Participants with a diagnosis of rapid cycling bipolar disorder had higher sexual abuse subscale scores than those who were not rapid cycling. In bipolar patients who reported one or more previous suicide attempts, CTQ total score was higher (*p =* 0.051), and scores significantly higher in the emotional abuse subscale.

**Table 3.** Analyses of bipolar group clinical severity and clinical characteristics.

	History of attempted suicide						Current suicidal ideation						Rapid cycling						DSM-IV melancholia					
	Yes *N* = 31^a^		No *N* = 24^a^		Comparison		Yes *N* = 14^a^		No *N* = 42^a^		Comparison		Yes *N* = 7^a^		No *N* = 48^a^		Comparison		Yes *N* = 25^a^		No *N* = 29^a^		Comparison	
	CTQ	(SD)	CTQ	(SD)	*U*	*p*	CTQ	(SD)	CTQ	(SD)	*U*	*p*	CTQ	(SD)	CTQ	(SD)	*U*	*p*	CTQ	(SD)	CTQ	(SD)	*U*	*p*
CTQ total	51.6	(23.1)	38.3	(12.8)	158.5	0.051	42.8	(19.4)	44.7	(20.3)	176.5	0.781	56.8	(32.6)	42.9	(16.3)	92.0	0.451	49.8	(20.8)	37.7	(14.9)	145.0	0.023
Emotional abuse	11.5	(5.9)	9.2	(5.0)	260.5	0.174	10.1	(5.6)	10.4	(5.6)	256.0	0.933	12.3	(6.4)	10.1	(5.3)	113.5	0.479	11.6	(6.2)	8.9	(4.2)	259.0	0.216
Physical abuse	8.3	(5.6)	6.9	(2.8)	322.5	0.831	6.8	(3.2)	7.9	(4.9)	234.0	0.569	10.2	(7.7)	7.2	(3.9)	116.0	0.503	7.8	(4.3)	7.0	(3.5)	264.5	0.227
Sexual abuse	9.0	(6.2)	6.7	(4.6)	265.0	0.198	8.4	(4.9)	7.7	(5.8)	190.0	0.184	12.7	(7.9)	7.4	(5.0)	75.5	0.038	8.2	(6.0)	7.6	(5.3)	319.0	0.907
Emotional neglect	13.7	(6.4)	11.4	(5.4)	255.0	0.146	9.7	(5.9)	13.1	(6.0)	169.0	0.058	12.5	(6.7)	12.6	(5.9)	129.5	0.807	14.3	(6.0)	10.9	(5.2)	210.0	0.031
Physical neglect	9.2	(4.3)	6.6	(2.7)	218.5	0.029	6.8	(2.2)	8.2	(4.3)	235.0	0.597	9.3	(6.5)	7.8	(3.5)	137.0	0.976	8.9	(3.7)	6.9	(3.8)	222.0	0.046

a Numbers vary due to the incomplete return of CTQs: history of attempted suicide, yes *N* = 23–29, no *N* = 21–23; current suicidal ideation, yes *N* = 11–13, no *N* = 34–40; rapid cycling, yes *N* = 6, no *N* = 38–46; DSM-IV melancholia, yes *N* = 21–24, no *N* = 23–27.

CTQ: Childhood Trauma Questionnaire.

No significant correlations between CTQ total or CTQ subscale scores and length of current episode, number of previous hospitalizations, current severity of depression (HDRS-17 score), current alcohol intake were found (*r*_s_ < 0.3, *p* > 0.1).

Bivariate correlations between pooled bipolar and control scores of the five trauma subscales found significant correlations between all subscales (0.33 < *r*_s_ < 0.64, *p <* 0.002), apart from between physical and sexual abuse (*r*_s_ = 0.12, *p =* 0.17). All correlations were below 0.8 and therefore could be entered into a regression model without risk of multi-colinearity. Differences or associations with CTQ subscale scores and demographic characteristics were limited to age, which was significantly, but weakly, correlated with emotional neglect (*r*_s_ = 0.14, *p =* 0.046) and sexual abuse scores which were significantly higher in females (*U* = 873.0, *p <* 0.001). NART scores or years of education were not significantly correlated with the CTQ subscales (*r*_s_ < 0.2, *p* > 0.1). The factors considered to be plausible independent causal risk factors, i.e. CTQ total score, age and sex, were entered into step forward logistic regression, with the dependent variable of group (bipolar or control). This confirmed CTQ total score was the only significant predictor (β = 0.08, *p =* 0.001). The five subscale scores, age and sex, were then entered into a second step forward logistic regression, also with the dependent variable of group. Emotional neglect (β = 0.185, *p* < 0.001) remained the only significant predictor in the model (Table 4). Emotional abuse approached significance (*p =* 0.082).

**Table 4.** Logistic regression of CTQ subtypes predicting a diagnosis of bipolar disorder (I and II).

	Score	df	*p*
Step 1			
Age	0.324	1	0.569
Gender	0.119	1	0.730
Emotional abuse	3.030	1	0.082
Physical abuse	1.790	1	0.181
Sexual abuse	0.017	1	0.896
Physical neglect	1.865	1	0.172

	β	Wald	*p*

Step 1^a^			
Emotional neglect	0.185	14.393	< 0.001
Constant	1.651	10.246	0.001

CTQ: Childhood Trauma Questionnaire.

## Discussion

This paper demonstrates significant associations between childhood trauma and bipolar disorder. Higher CTQ scores were found in patients diagnosed with both bipolar I and bipolar II disorder compared to controls. Sexual abuse was the only subscale measure that was not higher in bipolar patients compared with controls. In bipolar patients with a diagnosis of DSM-IV melancholia, emotional neglect and physical neglect scores were higher. CTQ subscale scores were higher in those with a past history of attempted suicide or a diagnosis of rapid cycling bipolar disorder. Logistic regression showed CTQ total scores to differentiate bipolar patients from controls, and separately identified emotional neglect to be the only significant subscale of the CTQ to differentiate bipolar patients from controls. Emotional abuse approached significance and may therefore be considered as a potential contributor to the model.

Our study is in line with the findings of two previous studies which also found that patients with a diagnosis of bipolar disorder reported higher rates of childhood trauma compared to healthy controls (Etain et al., 2010; Fowke et al., 2012). Exploring the subscales, we did not find significant differences in the sexual abuse scale, which is in accord with one previous report (Etain et al., 2010) and is supported by a recent paper that found sexual abuse to be the least reported form of abuse by bipolar patients (Larsson et al., 2013), although other studies did find this association (Fowke et al., 2012; Hyun et al., 2000). Our findings differed in identifying emotional neglect, as opposed to an earlier finding of emotional abuse, to be the single significant subscale associated with bipolar disorder (Etain et al., 2010; Fowke et al., 2012). Methodological differences with the previous studies utilising the CTQ (Etain et al., 2010; Fowke et al., 2012) relate to the presence or absence of current episode and to the sample size. Our finding that a history of childhood trauma is related to a history of suicide attempts in bipolar patients is also in line with other studies (Alvarez et al., 2011; Carballo et al., 2008; Garno et al., 2005; Leverich et al., 2002), although two of these studies did not use a validated measure to retrospectively assess for childhood trauma (Carballo et al., 2008; Leverich et al., 2002).

The allostatic impact of childhood trauma may be mediated through a range of biological systems with the hypothalamic–pituitary–adrenal (HPA) axis appearing to have a central role (Grande et al., 2012). It can also be argued that childhood trauma, at sensitive periods, may trigger an altered developmental pathway (Bateson et al., 2004), mediated in part by epigenetic processes (McGowan et al., 2009). For example, the regulation of hippocampal GR expression (McGowan et al., 2009) may induce ‘evolutionary appropriate’ responses such as increased vigilance, alertness to danger, responsivity to novel stressors and a willingness to explore new environments (Glover, 2011). The trade-off for such responses may be an increased risk of behavioural problems in childhood (Ramchandani et al., 2012) and of adult psychopathology including bipolar disorder (Watson et al., 2007) and suicidality (McGowan et al., 2009). It is of interest that emotional neglect was the only subscale which significantly differentiated patients from controls. Emotional neglect suggests a pervasive deficiency in the parent–child relationship (Glaser, 2002), has been repeatedly linked with HPA axis dysregulation in adults (Gerra et al., 2008, 2010; Watson et al., 2007) and has been previously shown to be differentially related to depression (Spinhoven et al., 2010).

It has been suggested that retrospective assessment of childhood trauma may be liable to recall bias in depressed patients (Lewinsohn and Rosenbaum, 1987). However, it should be noted that autobiographical recall of events (as measured using CTQ scores) in our study did not significantly correlate with severity of depression. CTQ scores have also been demonstrated to remain stable over time and to be independent of the current degree of abuse-related psychopathology (Paivio, 2001). Although there have been concerns that retrospective reporting overestimates associations between abuse and adult psychopathology compared to prospective assessment (Gilbert et al., 2009), a recent study found retrospective, compared to prospective, assessment of maltreatment predicted similar rates of mental disorder (Scott et al., 2012). A previous study has shown that recall bias accounted for less than 1% of reporting variance for measures of childhood abuse (Fergusson et al., 2011). However, emotional neglect is arguably the most subjective and difficult to define among forms of abuse, and hence further examination of the relationship between abuse and neglect and bipolar disorder in prospective studies which exclude recall bias would be useful. Investigations with euthymic bipolar patients would help to clarify the potential impact of current mood state. A weakness of this study is the relatively small sample size, which engenders the risk of type II errors. Further, the use of baseline data from a randomized controlled trial may have resulted in an under-sampling of more severe bipolar patients or those with comorbidities, which in turn may have resulted in an underestimation of the rates of childhood trauma in the bipolar group given the association between childhood trauma and poorer clinical outcomes (Garno et al., 2005; Leverich et al., 2002).

## Conclusions

The association of perceived childhood trauma and depression is established (Nanni et al., 2012). This study adds to the literature suggesting a similar relationship in bipolar disorder, although confirmation in prospective studies is desirable. Emotional neglect may be particularly pernicious. Further consideration of its psychological and neurobiological mediation is warranted.
